# Fully Parallel Implementation of Otsu Automatic Image Thresholding Algorithm on FPGA

**DOI:** 10.3390/s21124151

**Published:** 2021-06-17

**Authors:** Wysterlânya K. P. Barros, Leonardo A. Dias, Marcelo A. C. Fernandes

**Affiliations:** 1 Laboratory of Machine Learning and Intelligent Instrumentation, nPITI/IMD, Federal University of Rio Grande do Norte, Natal 59078-970, Brazil; kyurybarros@gmail.com; 2 Centre for Cyber Security and Privacy, School of Computer Science, University of Birmingham, Birmingham B15 2TT, UK; l.a.dias@bham.ac.uk; 3 Department of Computer and Automation Engineering, Federal University of Rio Grande do Norte, Natal 59078-970, Brazil

**Keywords:** FPGA, image segmentation, thresholding algorithm, Otsu’s method

## Abstract

This work proposes a high-throughput implementation of the Otsu automatic image thresholding algorithm on Field Programmable Gate Array (FPGA), aiming to process high-resolution images in real-time. The Otsu method is a widely used global thresholding algorithm to define an optimal threshold between two classes. However, this technique has a high computational cost, making it difficult to use in real-time applications. Thus, this paper proposes a hardware design exploiting parallelization to optimize the system’s processing time. The implementation details and an analysis of the synthesis results concerning the hardware area occupation, throughput, and dynamic power consumption, are presented. Results have shown that the proposed hardware achieved a high speedup compared to similar works in the literature.

## 1. Introduction

In recent years, there has been an increase in computational solutions employing Digital Image Processing (DIP) techniques, such as facial recognition, medical image enhancement, signature authentication, traffic control, autonomous cars, and product quality analysis [1,2,3,4,5]. These applications usually require real-time processing. However, meeting their processing time requirements can be complex due to the large volume of data to be processed, which is proportional to the image resolution, color depth, and, in the case of video applications, the frame rate employed. Therefore, obtaining results in real-time has become a challenge [6].

Some of the mentioned applications use segmentation algorithms to identify the image’s region of interest and classify its pixels as background or object. Thresholding is one of the main image segmentation techniques in which pixels are classified based on their intensity values [7]. The Otsu algorithm, proposed in [8], is a widely used global thresholding technique, which proposes the definition of an optimal threshold by maximizing the between-class variance. However, the Otsu algorithm has a high computational cost due to the complex arithmetic operations performed interactively, hindering its use in real-time applications.

Many works in the literature proposed the Otsu algorithm developed in hardware, such as Field-Programmable Gate Arrays (FPGA), to overcome the processing time constraints. This therefore allows applications to achieve real-time or near real-time processing. The FPGA allows the exploitation of the algorithm parallelization and the development of dedicated hardware to obtain performance improvement [9,10,11,12,13,14,15]. However, FPGA implementations found in the literature are often developed with sequential processing schemes in some stages of the Otsu algorithm, limiting the hardware’s processing speed [16,17,18,19,20,21].

Therefore, this work proposes a fully parallel FPGA implementation of the Otsu algorithm. Unlike most approaches proposed in the literature, a full-parallel implementation reduces the bottleneck for processing speed compared to sequential systems or hybrid hardware architectures, that is, architectures implemented with sequential and parallel schemes. Besides, given the continuous increase in the volume of data present in the DIP applications, a full-parallel strategy is less likely to become obsolete quickly.

The remainder of this paper is organized as follows: Section 2 presents the related works in the literature; Section 3 addresses the theoretical foundation of the Otsu method; Section 4 shows a detailed description of the architecture proposed in this paper; while Section 5 presents and analyzes the synthesis results obtained from the described implementation, including a comparison to other works. Finally, Section 7 presents the final considerations.

## 2. Related Works

Many proposals can be found in the literature for real-time applications of the Otsu algorithm deployed in FPGAs. In [22], an adaptive lane departure detection and alert system is presented, while in [23], a lane departure and frontal collision warning system. Meanwhile, in [24], a vision system is presented to detect obstacles and locate a robot that navigates indoors; in [25], a system for detecting moving objects is presented; [26], presents a system to assist in the diagnosis of glaucoma; and, in [27], a system for improving thermograms is presented. However, these articles provide few details about the hardware implementation.

Among the first FPGA implementations of the Ostu algorithm is the proposal of [16], synthesized for an Altera Cyclone II FPGA. The design improved the algorithm’s performance through a hybrid hardware architecture and Altera MegaCores, eliminating complex divisions and multiplications of the algorithm. The architecture developed was used for the segmentation of an image with a resolution of 320×240 and pixel represented by 10 bits. Through visual segmentation results, they evaluated the implementation performance as satisfactory.

Other works have proposed a hardware implementation using logarithmic functions to eliminate the division and multiplication circuits. In [17,19], the authors implemented two versions of the Otsu algorithm, in which one version uses the logarithmic functions, to compare the results achieved between them. The architectures developed by [17] were synthesized for a Xilinx Virtex XCV800 HQ240-4 FPGA. The implementation without the logarithmic functions occupied a hardware area of 622 slices and 103 Input/Output Blocks (IOBs), obtaining a clock latency of 362.4 ns. Meanwhile, the logarithmic function implementation occupied 109 slices and 49 IOBs, obtaining a clock latency of 132ns.

The architectures presented by [19] were developed on the Altera Cyclone IV EP4CE115F29C6N FPGA. The synthesis results obtained for the algorithm implemented without the logarithmic functions occupied 6525 logic elements, 4920 registers, 18,266 bits of memory, and 79 multipliers of 9 bits. Regarding the processing time, considering an image with a resolution of 1280×1024, the system achieved a maximum frequency of 130.89 MHz and latency of 589 clock cycles. In contrast, the implementation with the logarithmic function used 2440 logic elements, 1026 registers, 10,943 bits of memory, and 79 multipliers of 9 bits. Moreover, it achieved a maximum frequency of 114.62 MHz and a latency of 536 clock cycles. Therefore, the results presented in [17,19] indicate that the algorithm designed with logarithmic function reduced the FPGA area occupation and latency.

In [18,20], similar architectures of the Otsu algorithm in the Virtex-5 xc5vfx70t ffg1136-1 FPGA were deployed, available on the Xilinx ML-507 development platform. Both proposals were developed in VHDL, using fixed-point representation, operating at a clock frequency of 25.175 MHz. The proposal described in [18] occupied 168 slices and 33 IOBs, while in [20], the implementation reached an area occupation of 161 slices, 21 IOBs, 72 Look-Up Tables (LUTs), 591 registers, 4 blocks of RAM (BRAMs), and 5 DSP48Es. In addition, Reference [20] presented results related to the processing time for a 640×480 image, with pixels represented by 8 bits. This work reached a latency of 5 clock cycles and throughput of 40.28 megabits processed per second (Mbps).

Meanwhile, it was presented in [21] an implementation for binarization and thinning of fingerprint images, using the Otsu method, on a Spartan 6 LX45 FPGA. Concerning the area occupation, 1898 registers, 1859 LUTs, 735 slices, 10 IOBs and 44 BRAMs were used. Regarding processing time, a maximum clock frequency of 100MHz was achieved, with the execution time of 1489 ms for processing a 280×265 image and latency of 531 clock cycles or 5310 ns. Besides, a comparison with the same technique implemented in Matlab was also presented, showing that the FPGA was ≈10× faster than the Matlab version.

Thus, this work proposes an FPGA implementation of the Otsu algorithm to improve its performance. Unlike the works presented in the literature, the architecture proposed here uses a fully parallel scheme. The hardware implementation was developed in *Register-Transfer Level* (RTL), using fixed-point representation, in an Arria 10 GX 1150 FPGA. The results concerning the hardware area occupation and throughput are also presented.

## 3. Otsu’s Algorithm

The Otsu is one of the most popular thresholding algorithms, used to find an optimal threshold that separates an image into two classes: the background and object. These classes are represented by $C_{0}$ and $C_{1}$, respectively. This method has the advantage of performing all its calculations based only on the histogram of the image [7,8].

Initially, the algorithm starts by calculating the normalized histogram of an image, A, in grayscale, which is described as

(1) $$A\left(m\right)=\left[\begin{array}{ccccc} a_{0,0}\left(n\right) & \cdots & a_{0,j}\left(n\right) & \cdots & a_{0,M-1}\left(n\right)\\ \vdots & \ddots & \vdots & \ddots & \vdots \\ a_{i,0}\left(n\right) & \cdots & a_{i,j}\left(n\right) & \cdots & a_{i,M-1}\left(n\right)\\ \vdots & \ddots & \vdots & \ddots & \vdots \\ a_{N-1,0}\left(n\right) & \cdots & a_{N-1,j}\left(n\right) & \cdots & a_{N-1,M-1}\left(n\right) \end{array}\right]$$

where $a_{i,j}$ is one pixel of *b* bits and M×N is the image dimension. The pixels can assume *L* distinct integer intensity levels, represented by *k* and characterized as a value in a range of 0 to L−1, where $L=2^{b}$. Each *n*-th pixel is processed in an instant $t_{s}$, which represents the sampling time. Thus, one complete image can be processed at every *m*-th moment, where

(2) $$m=M\times N\times t_{s}.$$

This equation must be changed if more than one pixel is processed per sample time.

The histogram of each *m*-th image, A(m), is calculated and stored in the vector

(3) $$p\left(m\right)=[p_{0}\left(m\right),\ldots ,p_{k}\left(m\right),\ldots ,p_{L-1}\left(m\right)]$$

where each *k*-th component, $p_{k}\left(m\right)$, is defined as

(4) $$p_{k}\left(m\right)=\frac{n_{k}\left(m\right)}{M\times N},$$

in which $n_{k}\left(m\right)$ denotes the number of pixels with intensity *k* of the *m*-th image, described as

(5) $$n_{k}\left(m\right)=\sum_{i=0}^{N-1}\sum_{j=0}^{M-1}c_{i,j}\left(n\right),$$

with $c_{i,j}\left(n\right)$ expressed as

(6) $$c_{i,j}\left(n\right)=\left\{\begin{array}{cc} 1 & \;\mathrm{if}\;a_{i,j}\left(n\right)=k\\ 0 & \mathrm{otherwise} \end{array}.\right.$$

Subsequently, after obtaining the normalized histogram, stored in the vector p, the Otsu algorithm calculates an optimal threshold between the two classes, i.e., $C_{0}$ and $C_{1}$. The optimal threshold called here as $k^{*}\left(m\right)$ can be characterized as

(7) $$k^{*}\left(m\right)=\underset{0\leq k\leq L-1}{arg max}\sigma_{k}^{2}\left(m\right).$$

where $\sigma_{k}^{2}\left(m\right)$ is the *k*-th between-class variance of the *m*-th image, defined as

(8) $$\sigma_{k}^{2}\left(m\right)=\frac{{\left(\mu_{L-1}\left(m\right)\times \omega_{k}\left(m\right)-\mu_{k}\left(m\right)\right)}^{2}}{\omega_{k}\left(m\right)\left(1-\omega_{k}\left(m\right)\right)},$$

where $\omega_{k}\left(m\right)$ and $\mu_{k}\left(m\right)$ are the probability of class occurrence given a *k* threshold and the mean intensity value of the pixels up to the *k* threshold of the *m*-th image, respectively, meanwhile, $\mu_{L-1}\left(m\right)$ is the average intensity of the entire *m*-th image, called the global mean, with a value equal to $\mu_{k}\left(m\right)$ when k=L−1.

The variables $\omega_{k}\left(m\right)$ and $\mu_{k}\left(m\right)$ can be expressed as

(9) $$\omega_{k}\left(m\right)=\sum_{i=0}^{k}p_{i}\left(m\right)$$

and

(10) $$\mu_{k}\left(m\right)=\sum_{i=0}^{k}i\cdot p_{i}\left(m\right).$$

After finding the optimal threshold value, $k^{*}\left(m\right)$, the pixels of the input image,A(m), can be classified as a background or object ($C_{0}$ and $C_{1}$), generating a mask for the input image.

## 4. Hardware Proposal

This work proposes a fully parallel architecture of the Otsu method capable of processing images of any dimension, focused on obtaining high-speed processing. The details of the hardware implementation are described in the following subsections.

### 4.1. General Architecture

**Figure 1:**
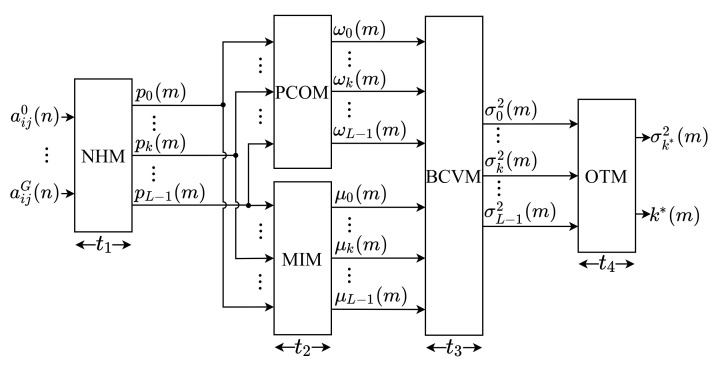
General architecture of the proposed Otsu Algorithm implementation.

The general hardware architecture implemented for the Otsu algorithm is presented through a block diagram, shown in Figure 1. As can be observed, the architecture was developed based on the description presented in Section 3. Therefore, it consists of five main modules: Normalized Histogram Module (NHM), Probability of Class Occurrence Module (PCOM), Mean Intensity Module (MIM), Between-Class Variance Module (BCVM), and Optimal Threshold Module (OTM).

Initially, the NHM module receives the parallel input of G image pixels, where G is the number of submodules internal to the NHM that simultaneously calculate the normalized histogram, according to Equations (3) and (4). Subsequently, the PCOM module uses the histogram components to calculate the class occurrence probabilities, according to Equation (9), while the MIM module calculates the average intensities, based on Equation (10). The PCOM and MIM modules perform their calculations simultaneously. Afterward, these two modules’ outputs are supplied to BCVM, in which the values of the between-class variance are computed, according to Equation (8). Finally, the calculated between-class variances are compared in the OTM to select the optimal threshold value, as described in Equation (7).

All variables and constants shown in Figure 1 were implemented in fixed point to reduce the bit-width compared to floating-point implementations, the bits number used can be adjusted to adapt the precision of the results obtained to the desired application. Therefore, each pixel, $a_{ij}^{g}\left(n\right)$, of the input image were configured with 8 bits in the integer part (without sign), then $L=2^{8}=256$ is defined. For the histogram components, $p_{k}\left(m\right)$, and the probabilities of class occurrence, $\omega_{k}\left(m\right)$, which has a positive value less than 1, only 24 bits are used in the decimal part. For the average intensity elements, $\mu_{k}\left(m\right)$, 8 bits are used in the integer part (without sign) and 24 bits in the decimal part. For the between-class variances, $\sigma_{k}^{2}\left(m\right)$, 27 bits are used in the integer part (one bit for sign) and 24 bits in the decimal part. Finally, for the optimal threshold, $k^{*}\left(m\right)$, only 8 bits are used in the integer part (without sign).

The modules of this architecture are pipelined, and the system operates on the same sample time, $t_{s}$. Nonetheless, each module has a different execution time, characterized here as $t_{1}$, $t_{2}$, $t_{3}$ and $t_{4}$. To minimize control, due to the lack of synchronism between the modules, the hardware proposed here defines $t_{1}=t_{2}=t_{3}=t_{4}=t_{I}$, where $t_{I}$ is the time to process a complete image, being equal to the *m*-th moment that an image is processed. The time $t_{I}$ is defined by the NHM block, since $t_{1}$ has the longest execution time. Thus, the system has an initial latency expressed as

(11) $$D=4\times t_{I}$$

and a throughput characterized as

(12) $$th=1/t_{I}.$$

### 4.2. Normalized Histogram Module (NHM)

**Figure 2:**
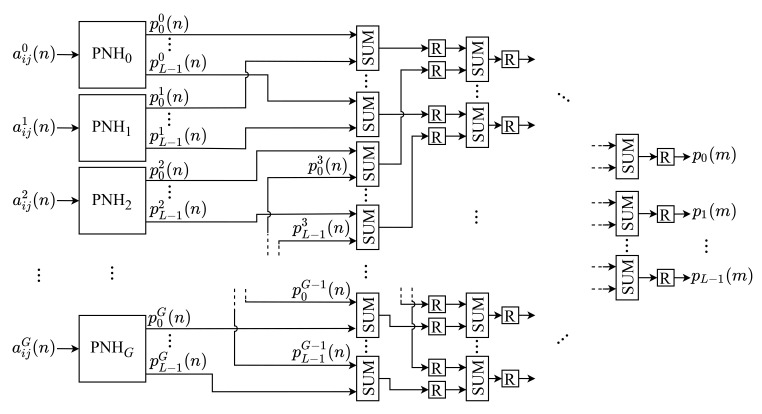
Architecture of the NHM.

The NHM is responsible for generating the normalized histogram of the input image, by performing the Equations (3) and (4). Usually, this step of the algorithm costs more clock cycles to complete than other steps, as the entire image needs to be scanned to obtain the histogram components. We propose the parallelization of this step by calculating the components’ partial values in a parallel way to optimize this process. Afterward, these values are summed to obtain their final values. The architecture of this module is shown in Figure 2.

**Figure 3:**
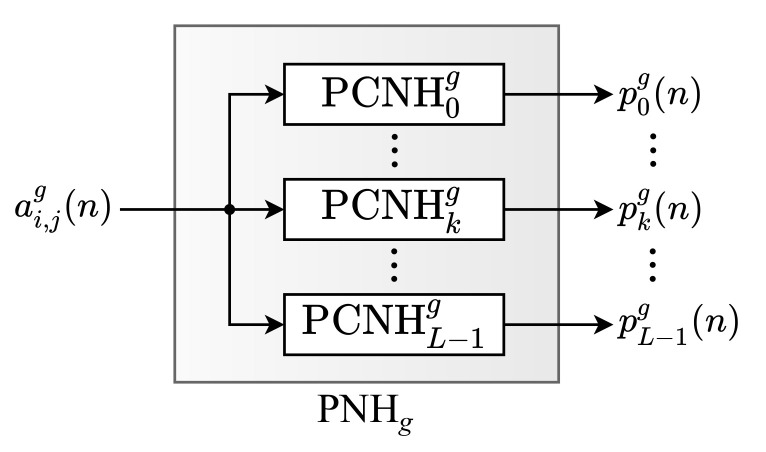
Architecture of the ${\mathrm{PNH}}_{g}$.

**Figure 4:**
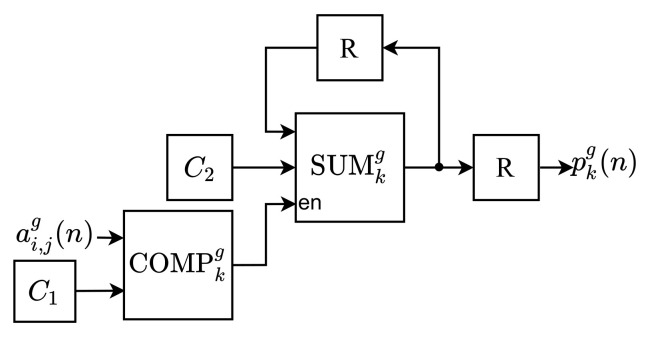
Architecture of the ${\mathrm{PCNH}}_{k}^{g}$.

As can be observed, the NHM module is constituted of *G* identical submodules, called Partial Normalized Histogram (PNH), responsible for computing the partial values of the histogram components. Each *g*-th input pixel, $a_{ij}^{g}\left(n\right)$, is processed by the *g*-th ${\mathrm{PNH}}_{g}$. Likewise, the PNH modules are internally constituted of *L* submodules, called Partial Component of the Normalized Histogram (PCNH), as shown in Figure 3. Thus, each *k*-th partial component of the histogram calculated by the *g*-th PNH, $p_{k}^{g}\left(n\right)$, is computed in parallel by a ${\mathrm{PCNH}}_{k}^{g}$ submodule. Figure 4 shows the internal circuit of each PCNH module, consisting of a comparator ($COMP_{k}^{g}$), an adder ($SUM_{k}^{g}$), two registers (*R*) and two constants ($C_{1}$ and $C_{2}$).

Initially, in each *k*-th comparator of the *g*-th ${\mathrm{PCNH}}_{k}^{g}$, $COMP_{k}^{g}$, it is checked whether the input pixel, $a_{ij}^{g}\left(n\right)$, has value equal to $C_{1}$, according to Equation (6). The constant $C_{1}$ has a different value of *k* in each ${\mathrm{PCNH}}_{k}^{g}$ submodule. Following, the $COMP_{k}^{g}$ output, $c_{i,j}\left(n\right)$, which is a Boolean value, enables the adder, $SUM_{k}^{g}$, when equal to 1. Therefore, when $SUM_{k}^{g}$ is enabled, the constant $C_{2}$ is summed with its previous value, thus operating as an accumulator. The constant $C_{2}$ is defined as $\frac{1}{M\times N}$, so it determines the value of $p_{k}\left(m\right)$ when summed $n_{k}$ times, according to Equation (4). After entering all the image pixels, each ${\mathrm{PCNH}}_{k}^{g}$ outputs the *k*-th partial component of the normalized histogram computed in the *g*-th PNH, $p_{k}^{g}\left(n\right)$.

After that, the final value of each *k*-th component of the *m*-th image, $p_{k}\left(m\right)$, is obtained by summing all the *k*-th partial values provided as an output of each *g*-th PNH, $p_{k}^{g}\left(n\right)$. This sum is performed for each *k*-th component through an adder tree (SUM), as represented in Figure 2. This tree has a depth equal to $\log_{2}G$. At the end, the value of the components of the normalized histogram, $p_{k}\left(m\right)$, is obtained, according to Equation (3).

Instead of processing 1 pixel per sample time in the histogram, the proposed architecture allows processing *G* pixels in parallel. Consequently, the amount of clock cycles required in this step is reduced and, thus, the processing time of a complete image, $t_{I}$. Consequently, the latency is also reduced, and throughput increased. With this scheme, the value of $t_{I}$ can be defined by

(13) $$t_{I}=\left(\frac{M\times N}{G}+\left\lceil log_{2}\left(G\right)\right\rceil \right)\times t_{s}.$$

Through this equation, it is possible to observe that the higher the value of *G*, the better the performance obtained.

In each PCNH submodule, the constant $C_{1}$ assumes a grayscale value between 0 and L−1 and is represented with only 8 bits in the integer part (without sign). The constant $C_{2}$, which has a positive value less than 1, uses only 24 bits in the decimal part. This bit-width is also used for all the adders of the NHM module, as the normalized histogram components also assume positive values less than 1. All the *k*-th components of the histogram, $p_{k}\left(m\right)$, are transmitted in parallel to PCOM and MIM.

### 4.3. Mean Intensity Module (MIM)

**Figure 5:**
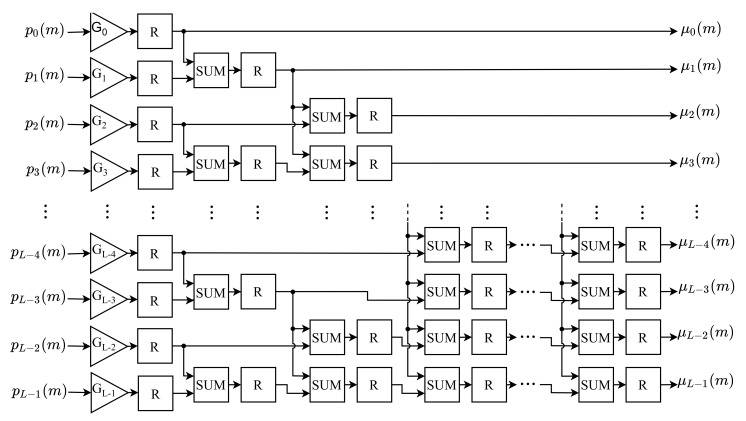
Architecture of the MIM.

The MIM calculates the average intensity value of the pixels up to level *k*, according to Equation (10). Each *k*-th average intensity, $\mu_{k}\left(m\right)$, is calculated in parallel. The architecture of this module, shown in Figure 5, consists of *L* gains submodules ($G_{k}$), $\frac{L}{2}\times \log_{2}L$ adders (SUM) and one register (*R*) after each component.

Based on the Equation (10), each *k*-th $p_{k}\left(m\right)$ component of the normalized histogram is first multiplied by a gain with the value of *k*. These gains are represented in the architecture block diagram by $G_{0},\ldots ,G_{L-1}$, and the index indicates the value of the applied gains. Thereafter, each *k*-th average intensity, $\mu_{k}\left(m\right)$, is obtained by summing the outputs of all gains with an index from 0 to *k*. The sum of these values is carried out in parallel based on the adder proposed by [28], with a maximum of $\log_{2}L$ cascading adders using this technique.

All *k*-th gains of this module, $G_{k}$, have their output represented with 8 bit in the integer part (without sign) and 24 bits in the decimal part. Similarly, this bit resolution is also used for the adders, SUM, as the average intensity values of the pixels are at most equal to L=256, for input images with pixels represented by 8 bits. All *k*-th average intensities, $\mu_{k}\left(m\right)$, are provided to BCVM in parallel.

### 4.4. Probability of Class Occurrence Module (PCOM)

The probability of class occurrence for a given threshold *k*, $\omega_{k}\left(m\right)$, is performed in PCOM based on Equation (9). This module has an architecture similar to MIM, but it does not have the gain submodule to weight the input. Therefore, the inputs are directly linked to the adders (SUM). Thus, this architecture is composed only of adders and registers, as shown in Figure 5.

According to Equation (9), the $\omega_{k}\left(m\right)$ values are obtained by adding all the components of the histogram from index 0 to *k*. Thus, using the parallel adder proposed by [28], all $\omega_{k}\left(m\right)$ values are computed simultaneously through the sum of the *k*-th entries $p_{k}\left(m\right)$.

The adders in this module were implemented for the same bit resolution of the inputs, $p_{k}\left(m\right)$, since the probability of class occurrence also assumes positive values less than 1. All *k*-th probabilities $\omega_{k}\left(m\right)$ calculated are propagated to the BCVM in parallel.

### 4.5. Between-Class Variance Module (BCVM)

**Figure 6:**
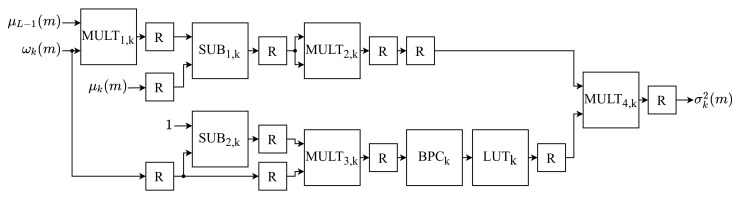
Architecture of the ${\mathrm{BCV}}_{k}$ submodule.

The *k*-th between-class variance of the *m*-th image, $\sigma_{k}^{2}\left(m\right)$, are calculated by BCVM based on the Equation (8). The BCVM module is internally composed of *L* equal submodules, named Between-Class Variance of *k* (${\mathrm{BCV}}_{k}$), with the same architecture shown in Figure 6. Each *k*-th $\sigma_{k}^{2}\left(m\right)$ is computed in parallel by the *k*-th submodule ${\mathrm{BCV}}_{k}$. This submodule consists of four multipliers ($MULT_{i,k}$), two subtractors ($SUB_{i,k}$), a point shift ($BPC_{K}$), a Look-Up Table ($LUT_{k}$) and eleven registers (*R*).

Equation (8) is performed in parallel in this architecture. Therefore, the numerator and denominator are performed simultaneously. Concerning the numerator, it is obtained by first multiplying the *k*-th probability, $\omega_{k}\left(m\right)$, by the global mean, $\mu_{L-1}\left(m\right)$, on $MULT_{1,k}$. Subsequently, the *k*-th $SUB_{1,k}$ submodule performs the subtraction between $\mu_{k}\left(m\right)$ and the $MULT_{1,k}$ output. Finally, the result of this subtraction is multiplied by itself in the *k*-th $MULT_{2,k}$. Regarding the denominator, it is calculated by initially subtracting the *k*-th probability, $\omega_{k}\left(m\right)$, from the value 1 in the *k*-th $SUB_{2,K}$. Lastly, the result is multiplied by the same $\omega_{k}\left(m\right)$ in the *k*-th $MULT_{3,k}$.

The division arithmetic operation is highly costly to the hardware in terms of processing speed, being the architecture’s bottleneck due to the highest critical time. One way to avoid using the division is to multiply the numerator by the reciprocal of the denominator. By definition, the reciprocal of a number is its inverse. Thereupon, the denominator’s reciprocal can be approximated for a range of predefined values and stored in a LUT. Thus, the division can be performed using only one LUT and a multiplier, $LUT_{k}$ and $MULT_{4,k}$, consequently increasing the throughput of the implementation.

Thus, each *k*-th value in the output of $MULT_{3,k}$ has a reciprocal approximated value in the *k*-th $LUT_{k}$. This LUT was configured with a depth of *L*, storing words of 33 bits, where 9 bits represent the integer part (one bit for sign) and 24 bits the fractional. The mapping of the output value of each *k*-th $MULT_{3,k}$ to an address of the $LUT_{k}$ is performed by shifting the binary point eight bits to the right by the *k*-th $BPC_{k}$. The approximate value of the reciprocal shown at the output of each *k*-th $LUT_{k}$ is multiplied by the calculated value of the numerator in $MULT_{4,k}$. The result of this multiplication is the *k*-th between-class variance of the *m*-th image, $\sigma_{k}^{2}\left(m\right)$.

The subtractor $SUB_{1,k}$ was configured with 9 bits in the integer part (one bit for sign) and 24 bits in the decimal part, while $SUB_{2,k}$, uses only 24 bits in the decimal part. The multiplier $MULT_{1,k}$ uses 8 bits in the integer part (without sign) and 24 bits in the decimal part, while $MULT_{2,k}$ uses 18 bits in the integer part (one bit for sign) and 24 bits in the decimal part. Meanwhile, $MULT_{3,k}$ was configured with only 24 bits in the decimal part, and $MULT_{4,k}$ uses 27 bits in the integer part (one bit for sign) and 24 bits in the decimal part. Each *k*-th between-class variance calculated is propagated in parallel to the OTM.

### 4.6. Optimal Threshold Module (OTM)

**Figure 7:**
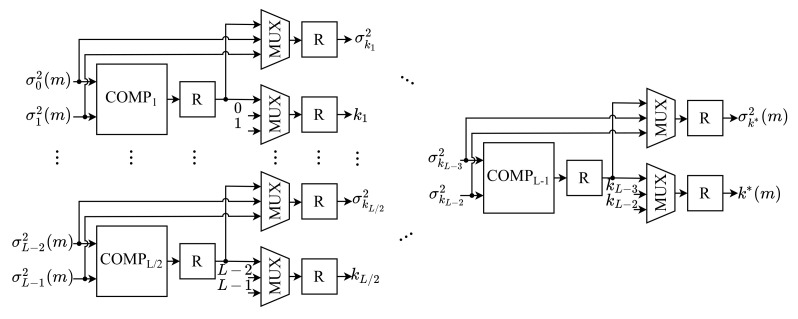
Architecture of the OTM.

The OTM module performs the last step of the Otsu algorithm, responsible for comparing all *k*-th values of the between-class variance, $\sigma_{k}^{2}\left(m\right)$, to determine the optimal threshold of the *m*-th image, $k^{*}\left(m\right)$, based on Equation (7). The architecture of this module is shown in Figure 7. As can be observed, it consists of L−1 comparators ($COMP_{k}$), 2×(L−1) multiplexers (MUX) and a register (*R*) after each component.

According to Equation (7), the optimal threshold of the *m*-th image, $k^{*}\left(m\right)$, is the threshold value for which the highest value of between-class variance is obtained. For this purpose, the between-class variances of the *m*-th image, $\sigma_{k}^{2}\left(m\right)$, are compared through a comparator tree. Each *k*-th $COMP_{k}$ compares whether a given variance $\sigma_{k}^{2}\left(m\right)$ is greater than the other, $\sigma_{k+1}^{2}\left(m\right)$. This comparator is used as a key selector of two multiplexers, defining on the outputs the largest variance value that was compared and its respective threshold *k*. All outputs of the multiplexers are passed to the next branch of the tree until the last comparator, $COMP_{L-1}$, in which the optimal threshold value of the *m*-th image, $k^{*}\left(m\right)$, should be selected as the output of the multiplexers, as well as its between-class variance, $\sigma_{k^{*}}^{2}\left(m\right)$.

Therefore, the optimal threshold of the *m*-th input image is determined by the proposed architecture of a fully parallel design. A new value of $k^{*}\left(m\right)$ is computed for every *m*-th instant.

## 5. Results

The architecture presented in the previous section was developed on an FPGA Arria 10 GX 1150, and the analysis of the synthesis results was carried out concerning hardware area occupation, throughput, and power consumption.

### 5.1. Hardware Area Occupation Analysis

Initially, the hardware area occupation analysis was performed for the architecture with one PNH module only. The results are shown in Table 1. The first to the third columns indicate the number of logical cells occupied ($n_{LC}$), the number of multipliers implemented using DSP blocks ($n_{Mult}$), and the number of block memory bits ($n_{BitsM}$), respectively. It also presents the resources used in percentage.

As can be observed, only 4% of the block memory bits available have been used, while 69% of the logic cells were occupied. The most-used resource was the multipliers, occupying 80% of the total DSPs available. Therefore, the data presented in Table 1 demonstrate the feasibility of implementing the proposed architecture in the target FPGA. Besides, the Arria-10 FPGA still has resources available that can be used to implement additional logic, thus allowing an increase in the number of PNH modules.

PNH modules require only logical cells for their implementation since they are not designed with multipliers and memories. Therefore, the 30% of unused logic cells in the Arria-10 allow for the increasing of the number of PNH modules. Hence, we also analyzed the area occupation for the Arria-10 FPGA by increasing the number of PNH modules ($n_{PNH}$). The number of occupied logical cells is presented in Table 2, as there is no change in the use of other resources.

**Figure 8:**
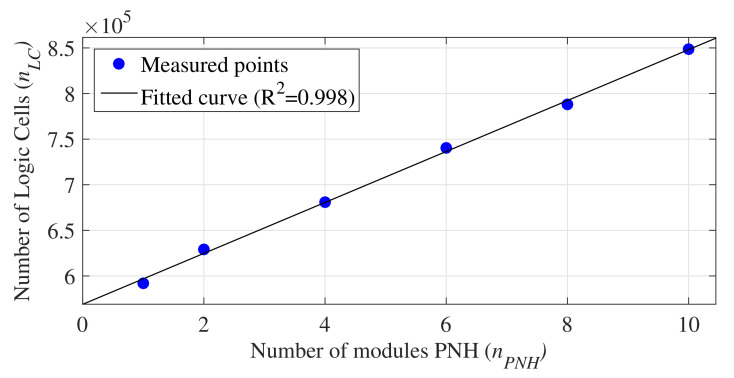
Linear regression curve for the hardware area occupation (logic cells) per $n_{PNH}$.

Figure 8 shows the curve obtained by linear regression using the set of values presented in Table 2. The equation associated with the regression analysis is expressed by

(14) $$n_{LC}=\left\lceil \left(2.8\times n_{PNH}+56.9\right)\times 10^{4}\right\rceil .$$

This equation can obtain the number of logical cells occupied by the architecture without the NHM module when defining $n_{PNH}=0$.

Therefore, Equation (14) allows for the estimation of the maximum number of PNH modules an FPGA can support. Table 3 presents the maximum number of PNH modules supported on different commercial FPGAs [29,30]. The first and second columns present the label and FPGA, respectively, while the third and fourth columns present the number of logical cells available ($n_{LC}^{max}$) and the maximum number of PNH modules that can be implemented with these resources ($n_{PNH}^{max}$). Hence, a high degree of parallelism can be achieved through our proposed architecture, limited only by the FPGA resources available.

### 5.2. Time Processing Analysis

The data related to the system’s processing time was obtained considering a clock cycle of 13.3 ns, which is defined by its critical path. Moreover, as the circuit operates with the same clock, the sampling time is also defined as $t_{s}=13.3$ ns.

The system’s processing time, for different amounts of $n_{PNH}$, is presented in Table 4. The first column indicates the number of $n_{PNH}$. Meanwhile, from the second to fourth columns are shown, respectively, the image processing time, $t_{I}$, defined according to Equation (13), the initial system latency, *D*, according to Equation (11) and the throughput, th, which in this work consists of the number of images processed per second (IPS), determined through Equation (12). According to Equation (13), the processing time of an image depends on its size. Thus, the data presented in Table 4 concern the processing of a 3840×2160 image with 4K resolution.

**Figure 9:**
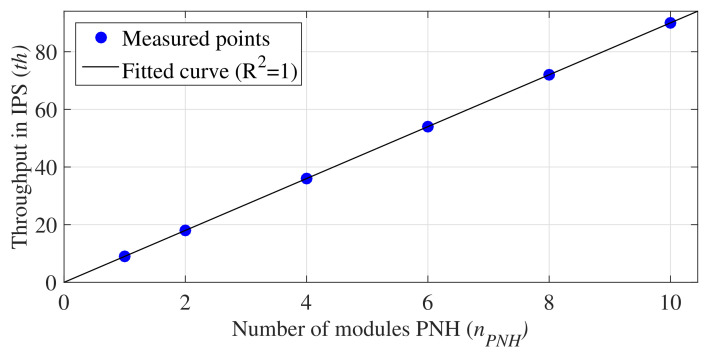
Throughput in IPS, th, per $n_{PNH}$.

As can be observed in Table 4, the value of $n_{PNH}$ is directly proportional to th and inversely proportional to $t_{I}$ and *D*. Thus, the more PNH modules employed in the implementation, the better the system performance. Figure 9 shows the curve obtained by linear regression that relates the values of $n_{PNH}$ and th. The equation associated with this curve is expressed by

(15) $$th=th\left(n_{PNH}=1\right)\times n_{PNH}.$$

According to (12), and (13), Equation (15) can be rewritten as

(16) $$th=\frac{1}{\left(\frac{M\times N}{1}+\left\lceil log_{2}\left(1\right)\right\rceil \right)\times t_{s}}\times n_{PNH}=\frac{n_{PNH}}{M\times N\times t_{s}}.$$

Equation (16), allows one to define $t_{I}$ values as

(17) $$t_{I}=\frac{1}{th}=\frac{M\times N\times ts}{n_{PNH}}.$$

When comparing this equation to Equation (13), it is observed that the only difference between them is the absence of the sum of the logarithm. The reason for that is

(18) $$\left\lceil log_{2}\left(n_{PNH}\right)\right\rceil \ll \frac{M\times N}{n_{PNH}}.$$

Thus, this component can be disregarded in the calculation of $t_{I}$.

Afterward, the proposed architecture’s processing time was analyzed for other commercial FPGAs, previously presented in Table 3, adopting for each FPGA an $n_{PNH}=n_{PNH}^{max}$. For this purpose, the values $t_{I}$ and th were defined according to Equations (16) and (17), respectively, and different image resolutions were considered. The results are shown in Table 5.

All FPGA models analyzed achieved a high throughput in processing images with 4 K resolution, allowing real-time processing of 4 K videos in all models. For images with 8 K and 10 K resolutions, real-time processing proved to be more viable in the FPGA_2_ and FPGA_3_. Finally, a high throughput was also achieved by the FPGA_3_ in processing 16 K images, allowing real-time processing of videos with this resolution. Therefore, the FPGA_3_ offers better performance due to the high number of PNH modules that can be implemented, i.e., the increased architecture’s parallelism.

## 6. Comparison with State-of-the-Art Works

Comparisons with the state-of-the-art works were performed for the three commercial FPGAs previously mentioned. The architectures were implemented using the maximum number of PNH modules, presented in Table 3. The results were compared only with works that presented data about hardware area occupation and processing time.

### 6.1. Hardware Occupation Comparison

Table 6 presents the comparison of the hardware area occupation. The first column indicates the reference analyzed. The second to fifth columns show the FPGA, the number of logical cells, multipliers, and the number of bits of memory blocks, respectively, of the reference. Meanwhile, the last four columns present the commercial FPGA to be compared and the ratio between the hardware components used in our proposed implementation, $n_{work}$, and those used in the literature, $n_{ref}$. The ratio of the hardware occupation can be expressed as

(19) $$R_{occupation}=\frac{n_{work}}{n_{ref}},$$

where $n_{work}$ and $n_{ref}$ can be replaced by $n_{LC}$, $n_{MULT}$, or $n_{BitsM}$. This ratio was calculated using the hardware occupancy data presented in Table 1 and Table 3.

The proposal presented by [21] was deployed on a Spartan-6 LX45 FPGA and occupied an area of 1859 LUTs and 44 RAM blocks. This FPGA uses about 1.6 logic cells (LC) per LUT, having used about 2,975 LC, and each block of RAM has 18 Kbits, so 792 Kbits of memory is used [31]. The number of multipliers used was not available.

In [19], results were presented for two different implementations of the Otsu method, deployed on the Altera Cyclone IV EP4CE115F29C6N FPGA. The comparison with this work was performed considering the best results presented by its authors, i.e., the method using logarithmic functions. The implemented hardware used 2440 LC, 10.94 Kbits of memory and 46 multipliers.

The proposal presented in [20] uses the Virtex-5 xc5vfx70tffg1-136-1 FPGA and occupied a total of 161 slices, 4 BRAMs and 5 multipliers. Each slice of this FPGA has 4 LUTs of 6-input, hence, 6.4 LC per slice was used, and each memory block has 36 Kbits [32]. Thus, about 1030 LC and 144 Kbits of memory were used.

Through Table 6, we found that in all comparisons performed our design presented a more significant hardware area occupation, due to the high degree of parallelism adopted in our proposed implementation, which results in more hardware resources.

### 6.2. Time Processing Comparison

Table 7 presents a throughput comparison. The analysis was carried out for all the commercial FPGAs previously presented. As can be seen, the first column presents the analyzed reference, while the second to fourth columns show the image resolution (IR), the clock (Clk), and the throughput achieved ($th_{ref}$), respectively, by the reference. The last three columns indicate, respectively, the FPGAs analyzed, the throughput ($th_{work}$), and speedup reached with our architecture. The values of $th_{work}$ are calculated according to Equation (16), employing the same clock and image size adopted by the compared reference. The speedup calculation is defined as

(20) $$Speedup=\frac{th_{work}}{th_{ref}}.$$

In [21], the runtime results are presented using a clock of 10 ns and a 280×265 input image, with pixels represented by 8 bits. The processing time is 1.489 ms, achieving a throughput of 671.59 IPS.

In [19], time and latency data were presented considering a clock of 8.72 ns and the processing of a 1280×1024 image, also with pixel representation for 8 bits. As the processing time of an image was not presented by the authors, it was estimated as the clock value times the number of cycles required to enter an image and obtain its respective output. The processed image’s input and output are performed serially, requiring 1280×1024 clocks cycles to scan the entire image. The latency indicated for the implementation of the Otsu method using logarithmic optimization is equal to 536. Thus, the calculated time for processing an image is equal to $\left(1280\times 1024\times 2+536\right)\times 8.72\times 10^{-9}\approx 22.87$ ms per image. Hence, achieving a throughput of 43.72 IPS.

The proposal presented by [20] displays results from throughput for processing a 640×480 image, with pixels represented by 8 bits, and adopting a clock of 39.72 ns. The throughput shown indicates the number of megabits processed per second (Mbps), which in turn is 40.28 Mbps. As each pixel has 8 bits, the number of images processed per second can be obtained by $\frac{40.28\;\times \;10^{6}}{8\;\times \;640\;\times \;480}\approx 16.39$ IPS.

According to the results presented in Table 7, our proposed architecture obtained better throughput values than other works in the literature in all comparisons performed. High speedup values were obtained, varying between 20× and 1128×. The performance achieved by the developed architecture is mainly due to the high degree of parallelism explored in this proposal, with a focus on parallelizing the calculation of the histogram. In contrast, works in the literature present implementations with a low degree of parallelism and are focused on optimizing the calculation of between-class variance.

### 6.3. Power Consumption Comparison

Table 8 presents a comparison of our design’s dynamic power consumption compared to other proposals in the literature. The dynamic power consumption can be expressed as

(21) $$P_{d}\propto N_{g}\times F_{clk}\times V_{DD}^{2}$$

where $P_{d}$ is the dynamic power consumption, $N_{g}$ is the number of hardware components, $F_{clk}$ is the frequency and $V_{DD}$ is the supply voltage. The frequency at which a circuit can operate is proportional to the voltage [12,33]. Thus, the Equation (21) can be rewritten as

(22) $$P_{d}\propto N_{g}\times F_{clk}^{3}.$$

For all comparisons, the value of $N_{g}$ was calculated as

(23) $$N_{g}=n_{LC}+n_{Mult}.$$

Based on the Equation (22), the saved dynamic energy, $S_{d}$, can be expressed by

(24) $$S_{d}=\frac{N_{g}^{ref}\times {\left(F_{clk}^{ref}\right)}^{3}}{N_{g}^{work}\times {\left(F_{clk}^{work}\right)}^{3}}$$

where $N_{g}^{ref}$ and $F_{clk}^{ref}$ are the number of hardware components and clock of literature works, respectively, and $N_{g}^{work}$ and $F_{clk}^{work}$ are the number of components and the clock adopted in this work to obtain the same throughput of the reference to which it is being compared.

Table 8 shows that our implementation presents a significant reduction in energy consumption. The results presented indicate an energy saving, regarding works in the literature, between $2.33\cdot 10^{1}$ and $9.98\cdot 10^{5}\times$. This reduction is obtained through the high degree of parallelism of the technique, which allows one to obtain high throughput with the circuit operating at a low clock frequency. Therefore, although the circuit uses many hardware components, the energy consumed is reduced due to the low-frequency operation.

## 7. Conclusions

This work presented a parallel implementation proposal of the Otsu method in FPGA. The hardware architecture was developed using RTL design and fixed-point representation. All the implementation details were presented, and the synthesis results were related to the hardware area occupation and processing time. The results showed that the proposed architecture achieved high throughput, enabling real-time processing of high-resolution videos. Comparisons with state-of-the-art works were also performed regarding the hardware area occupancy, throughput and dynamic power consumption. Our proposed architecture outperformed the compared ones in terms of throughput and power consumption, achieving a speedup from 20× until $10^{3}\times$ and reducing the power from 23× until $10^{5}\times$. However, the amount of hardware resources required is higher than in other architectures due to the high degree of parallelism adopted in our method. Finally, as future work, a study will be carried out to analyze the impact on the result’s accuracy by reducing the number of bits used. Besides, tests will be conducted with the processing of videos in real-time.

## Figures and Tables

**Table 1:** Hardware area occupation for $n_{PNH}=1$.

$n_{\mathrm{LC}}$	$n_{\mathrm{Mult}}$	$n_{\mathrm{BitsM}}$
591,946 (69.3%)	1222 (80.5%)	2162 K (3.9%)

**Table 2:** Number of logic cells required per PNH module.

$n_{\mathrm{PNH}}$	$n_{\mathrm{LC}}$
1	591,946 (69.3%)
2	629,096 (73.6%)
4	680,884 (79.7%)
6	740,368 (86.6%)
8	788,092 (92.2%)
10	848,630 (99.3%)

**Table 3:** Estimated number of PNH modules, $n_{PNH}^{max}$, for some commercial FPGAs.

Label	FPGA	$n_{\mathrm{LC}}^{\max}$	$n_{\mathrm{PNH}}^{\max}$
FPGA_1_	Arria 10 GX 1150	854,400	10
FPGA_2_	Agilex AGF 027	1,825,600	44
FPGA_3_	Stratix 10 GX 10M	6,932,160	227

**Table 4:** System’s processing time.

$n_{\mathrm{PNH}}$	$t_{I}$ (ms)	*D* (ms)	th (IPS)
1	110.32	441.28	9
2	55.16	220.64	18
4	27.58	110.32	36
6	18.39	73.56	54
8	13.79	55.16	72
10	11.03	44.12	90

**Table 5:** Values of $t_{I}$ and th for some commercial FPGAs for different image sizes.

Image Size	FPGA_1_		FPGA_2_		FPGA_3_	
	$t_{I}$ (ms)	th (IPS)	$t_{I}$ (ms)	th (IPS)	$t_{I}$ (ms)	th (IPS)
4 K (3840×2160)	11.03	90	2.51	398	0.49	2057
8 K (7680×4320)	44.13	22	10.03	99	1.94	514
10 K (10,240×4320)	58.80	16	13.40	74	2.60	385
16 K (15,360×8640)	176.50	5	40.11	24	7.78	128

**Table 6:** Hardware occupation comparison with other works.

Ref.	FPGA_ref_	$n_{\mathrm{LC}}$	$n_{\mathrm{Mult}}$	$n_{\mathrm{BitsM}}$	FPGA_work_	R_occupation_		
						$n_{\mathrm{LC}}$	$n_{\mathrm{Mult}}$	$n_{\mathrm{BitsM}}$
[21]	Spartan 6	2975	-	792 K	FPGA_1_	≈287.19×	-	≈2.73×
					FPGA_2_	≈613.65×	-	≈2.73×
					FPGA_3_	≈2330.13×	-	≈2.73×
[19]	Cyclone IV	2440	46	10.94 K	FPGA_1_	≈350.16×	≈26.56×	≈197.62×
					FPGA_2_	≈748.20×	≈26.56×	≈197.62×
					FPGA_3_	≈2841.05×	≈26.56×	≈197.62×
[20]	Virtex 5	1031	5	144 K	FPGA_1_	≈828.71×	≈244.40×	≈15.01×
					FPGA_2_	≈1770.71×	≈244.40×	≈15.01×
					FPGA_3_	≈6723.72×	≈244.40×	≈15.01×

**Table 7:** Throughput comparison with other works.

Ref.	RI	Clk (ns)	${\mathrm{th}}_{\mathrm{ref}}$ (IPS)	FPGA	${\mathrm{th}}_{\mathrm{work}}$ (IPS)	Speedup
[21]	280×265	10.00	671.59	FPGA_1_	13,469.83	≈20.06×
				FPGA_2_	59,088.95	≈87.98×
				FPGA_3_	298,621.34	≈444.65×
[19]	1280×1024	8.72	43.72	FPGA_1_	874.90	≈20.01×
				FPGA_2_	3848.92	≈88.03×
				FPGA_3_	19,833.44	≈453.65×
[20]	640×480	39.72	16.39	FPGA_1_	819.43	≈49.99×
				FPGA_2_	3602.87	≈219.82×
				FPGA_3_	18,494.20	≈1128.38×

**Table 8:** Dynamic power comparison with other works.

Ref.	$N_{g}^{\mathrm{ref}}$	$F_{\mathrm{clk}}^{\mathrm{ref}}$ (MHz)	$N_{g}^{\mathrm{work}}$	FPGA	$F_{\mathrm{clk}}^{\mathrm{work}}$ (MHz)	$S_{d}$
[21]	2975	100.00	593,168	FPGA_1_	4.98	≈$2.82\cdot 10^{1}\times$
				FPGA_2_	1.13	≈$1.13\cdot 10^{3}\times$
				FPGA_3_	0.22	≈$4.03\cdot 10^{4}\times$
[19]	2486	114.62	593,168	FPGA_1_	5.73	≈$2.33\cdot 10^{1}\times$
				FPGA_2_	1.30	≈$9.33\cdot 10^{2}\times$
				FPGA_3_	0.25	≈$3.46\cdot 10^{4}\times$
[20]	1036	25.175	593,168	FPGA_1_	0.50	≈$1.55\cdot 10^{2}\times$
				FPGA_2_	0.11	≈$6.80\cdot 10^{3}\times$
				FPGA_3_	0.02	≈$9.98\cdot 10^{5}\times$
